# Identification of a novel partner gene, *KIAA1217*, fused to *RET*: Functional characterization and inhibitor sensitivity of two isoforms in lung adenocarcinoma

**DOI:** 10.18632/oncotarget.9137

**Published:** 2016-05-02

**Authors:** Mi-Sook Lee, Ryong Nam Kim, Hoseok I, Doo-Yi Oh, Ji-Young Song, Ka-Won Noh, Yu-Jin Kim, Jung Wook Yang, Maruja E. Lira, Chang Hun Lee, Min Ki Lee, Yeoung Dae Kim, Mao Mao, Joungho Han, Jhingook Kim, Yoon-La Choi

**Affiliations:** ^1^ Samsung Advanced Institute for Health Sciences and Technology, Sungkyunkwan University School of Medicine, Seoul, Korea; ^2^ Laboratory of Cancer Genomics and Molecular Pathology, Samsung Biomedical Research Institute, Samsung Medical Center, Seoul, Korea; ^3^ Department of Pharmacy, College of Pharmacy, Seoul National University, Seoul, Korea; ^4^ Tumor Microenvironment Global Core Research Center, Seoul National University, Seoul, Korea; ^5^ Department of Thoracic and Cardiovascular Surgery, Pusan National University School of Medicine, Busan, Korea; ^6^ Pusan National University Hospital Biomedical Research Institute, Busan, Korea; ^7^ Pfizer Oncology, San Diego, California, USA; ^8^ Department of Pathology, Pusan National University School of Medicine, Busan, Korea; ^9^ Department of Pulmonology Allergy and Critical Care Medicine, Pusan National University School of Medicine, Busan, Korea; ^10^ Departments of Pathology and Translational Genomics, Samsung Medical Center, Sungkyunkwan University College of Medicine, Seoul, Korea; ^11^ Department of Pathology, Gyeongsang National University School of Medicine, Jinju, Korea; ^12^ Department of Thoracic Surgery, Samsung Medical Center, Sungkyunkwan University College of Medicine, Seoul, Korea; ^13^ Present address: BGI Genomics, Shenzhen, China

**Keywords:** lung cancer, RET, KIAA1217, KIAA1217-RET fusion, oncogenic driver

## Abstract

REarranged during Transfection (*RET*) fusion genes are detected in approximately 1% of lung adenocarcinomas and known primarily as oncogenic driver factors. Here, we found a novel *RET* fusion gene, *KIAA1217-RET*, and examined the functional differences of RET51 and RET9 protein, fused with KIAA1217 in cancer progression and drug response. *KIAA1217-RET*, resulting from the rearrangement of chromosome 10, was generated by the fusion of *KIAA1217* exon 11 and *RET* exon 11 from a non-small cell lung cancer patient. Expression of this gene led to increased cell growth and invasive properties through activations of the PI3K/AKT and ERK signaling pathways and subsequently enabled oncogenic transformation of lung cells. We observed that cells expressing KIAA1217-RET9 fusion protein were more sensitive to vandetanib than those expressing *KIAA1217-RET51* and both isoforms attenuated cellular growth via cell cycle arrest. These results demonstrated that *KIAA1217-RET* fusion represents a novel oncogenic driver gene, the products of which are sensitive to vandetanib treatment, and suggested that the *KIAA1217-RET*-fusion gene is a promising target for lung cancer treatment.

## INTRODUCTION

Recent advances in sequencing technology enabled the comprehensive detection of rearrangements in the cancer genome and transcriptome [1]. Chromosomal rearrangements involving receptor tyrosine kinases (RTKs) are an important class of cancer-related somatic variation and have emerged as oncogenic drivers in cancer progression [2]. More importantly, a number previous reports, describing the oncogenic behavior and therapeutic response of fusion genes in selected groups have shown their clinical benefits [3, 4]. For example, patients having *EML4-ALK* fusion in non-small-cell lung cancer (NSCLC) are highly responsive to the ALK inhibitor crizotinib and ceritinib [5]. The success of crizotinib reinforces the importance identifying of the oncogenic fusion genes and assessing their sensitivity to therapeutics.

The rearranged-during-transfection (*RET*) proto-oncogene is located in the pericentromeric region of chromosome 10q11.2 and encodes a single-pass transmembrane RTK [6]. The *RET* rearrangement has been identified in approximately 1–2% of NSCLC patients [7–9]. To date, genomic rearrangement of *RET* combined with kinesin family member 5B (*KIF5B*) has been identified with more than 10 variations, displaying a variety of breakage positions within the *KIF5B* locus [3, 10]. Additionally, five other *RET* fusion partner genes have been identified: *CCDC6* (coiled-coil domain containing 6), *CUX1* (cut-like homeobox 1), *TRIM33* (tripartite-motif containing 33), *NCOA4* (nuclear-receptor coactivator 4), and *KIAA1468* [11–15]. The transformation potential of the *KIF5B-RET* or *CCDC6-RET* fusions has been reported in Ba/F3 cells and LC-2/ad, the human lung-adenocarcinoma cell line [9, 11, 16]. Similar results were observed in NIH3T3 cells displaying anchorage-independent cell proliferation [9, 17, 18].

RET tyrosine kinase is generally expressed at very low levels in normal lung but, oncogenic *RET* is activated by point mutations within its tyrosine kinase domain or genomic rearrangements that produce chimeric RET proteins. These RET fusion proteins frequently contain coiled-coil domains (CCDs) within their partner genes and result in aberrant activation of RET kinase by their CCD-dependent homodimerization [2, 19, 20]. RET dimerization and autophosphorylation via intracellular tyrosine residues 1062 (pY1062) and pY1096 (in the RET51 isoform only) recruit adaptor and signaling proteins to stimulate multiple downstream molecules [6]. Consequently, signaling pathways related to cell proliferation and survival are activated, including the phosphoinositide 3-kinase (PI3K)/AKT, extracellular-signal regulated kinase (ERK)/mitogen-activated protein (MAP) kinase, and STAT3 pathways [21–24].

*KIAA1217* [also known as *SKT*, the human homolog of murine *Skt* (*Sickle tail*)], the novel *RET*-fusion partner gene identified in this study, is required for normal development of intervertebral disks, and is ubiquitously expressed in the cytoplasm. The *KIAA1217* gene that fuses to *RET* kinase has two CCDs and can induce aberrant activation of RET kinase through CCD-dependent dimerization. Here, we discovered a novel *KIAA1217-RET* fusion in NSCLC and demonstrated its potential biological significance as an oncogenic driver and pro-invasive gene using both *in vivo* and *in vitro* assays. Furthermore, we described the effects of vandetanib in RET fusion-positive lung cells.

## RESULTS

### Identification of the *KIAA1217-RET* fusion gene

**Figure 1:**
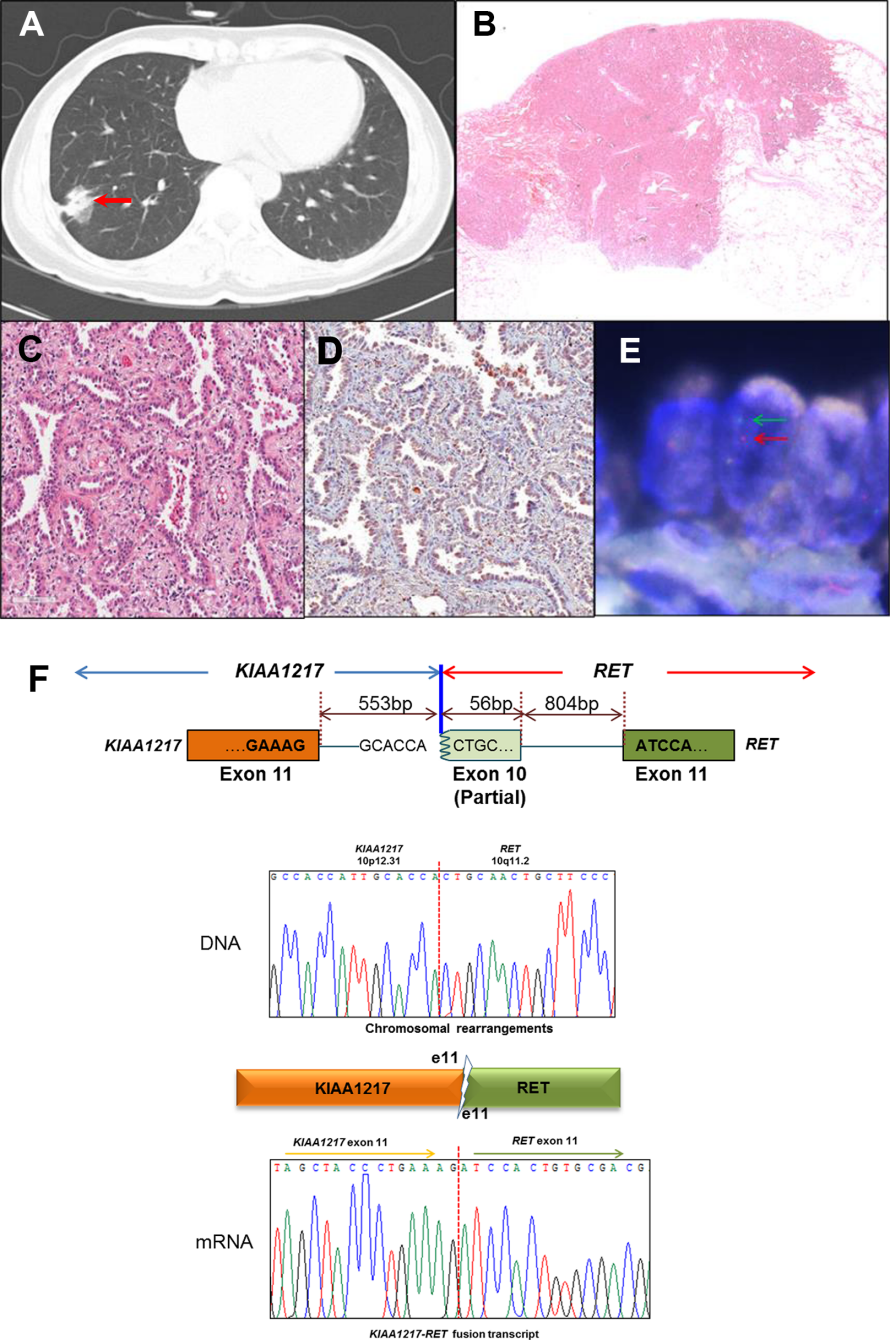
Identification of the *KIAA1217-RET* fusion gene, (A–E) Clinical and pathological analysis of lung adenocarcinoma harboring *RET* fusion genes (A) A 4-cm solid tumor nodule (red arrow) on the right lower lobe shown by chest computed-tomography scan. (B and C) Histologic features of lung adenocarcinoma harboring *RET* rearrangement. Adenocarcinoma with a predominant acinar pattern in hematoxylin-and-eosin staining (4 × and 200 ×). (D) Immunohistochemistry of RET shows both membrane and cytoplasm localization (200 ×). (E) Fluorescence *in situ* hybridization analysis. The split signals (5′-red and 3′-green) were detected in tumor cells. (**F**) The breakpoints in the *KIAA1217-RET* fusion gene were identified at the genomic and transcript levels by Sanger sequencing from patient T-#261.

Previously, we screened for *ALK, ROS1*, and *RET* fusion genes in 795 lung adenocarcinoma specimens and identified the novel *KIAA1217-RET* fusion gene containing *KIAA1217* exon 11 and *RET* exon 11 [7, 25] from a patient with a 4-cm tumor mass (Figure 1A, red arrow). Hematoxylin and -eosin staining revealed an adenocarcinoma with a predominant acinar pattern (Figure 1B and 1C), and the immunohistochemistry results showed that the RET protein was mainly localized in the cytoplasm (Figure 1D). In most cell types, RET proteins localize at the plasma membrane [21] however, RET fusion proteins could be changed in its localization depending on their partner gene such as *CCDC6* and *NCOA4,* which are localized in cytoplasm [7]. The RET fusion was confirmed by fluorescence *in situ* hybridization (FISH) analysis, resulting a split between the 5′- and 3′-RET signals (Figure 1E, green and red arrows). The tumor did not harbor either *EGFR or*, *KRAS* mutations or *ALK* rearrangement.

**Figure 2:**
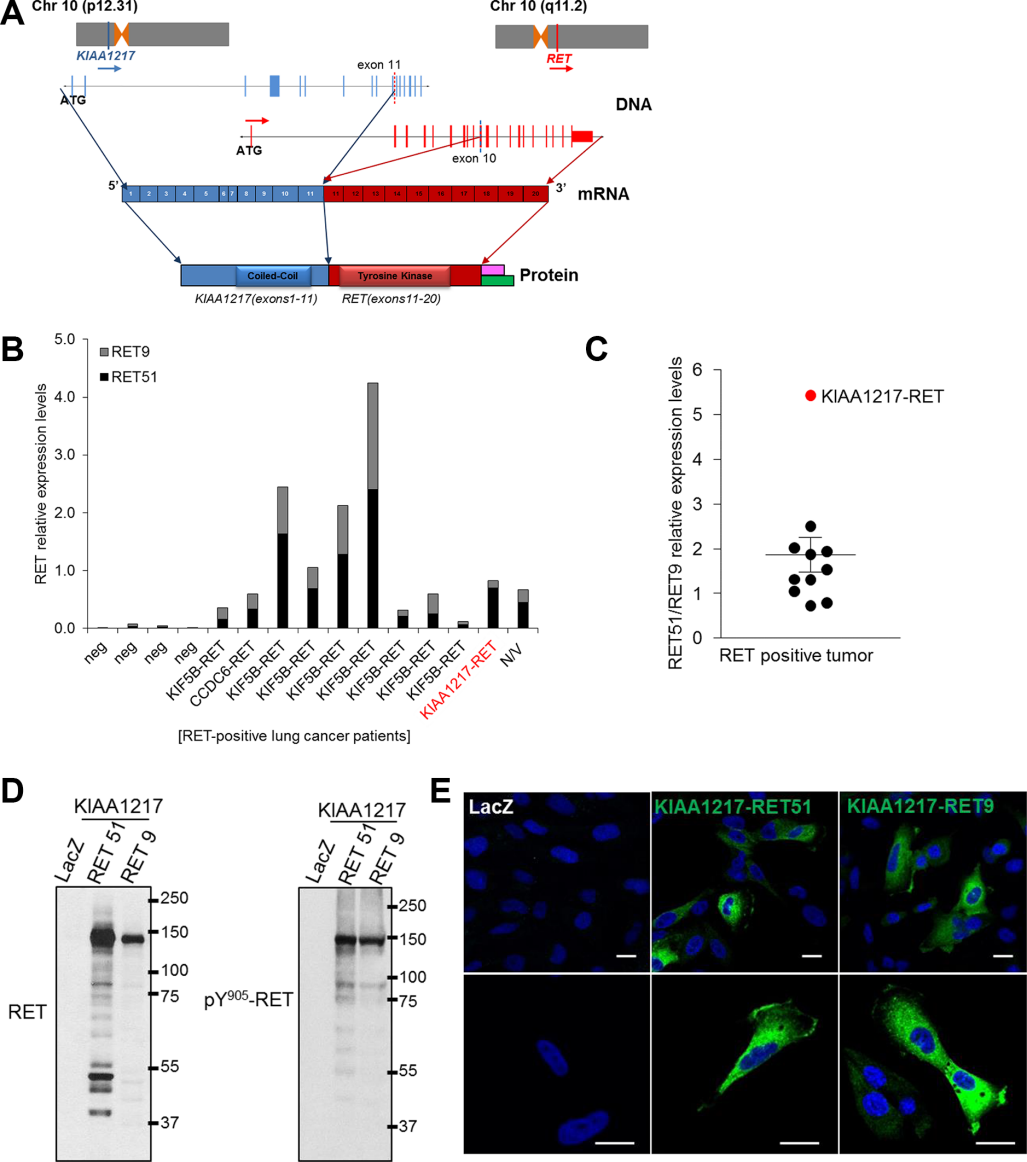
*KIAA1217-RET* fusion genes (**A**) Schematic representation of the predicted *KIAA1217-RET* fusion genes identifying the conjoined region in the genome and the transcript by Sanger sequencing. Pink: *RET9*, Green: *RET51,* (**B** and **C**) Identification of RET isoforms from RET fusion-positive lung cancer patients. (B) The relative mRNA levels of total RET and RET isoforms were analyzed using qRT-PCR and calculated with ΔΔCt values. The values of RET51 and RET9 shown in the graph are presented as ratios of RET51 and RET9 to total RET mRNA. The values shown represent the average of triplicate experiments. Lung cancer patients not harboring the RET fusion are labeled as “neg”, and “N/V” denotes a RET-fusion-positive patient in which a partner gene was not identified (C) The RET51/RET9 relative expression levels were presented as fold changes of relative ΔΔCt values obtained from qRT-PCR and each dot represents relative ratio of RET51/RET9 mRNA expression per patient with RET fusion. (**D**) The *KIAA1217-RET51* or *KIAA1217-RET9* fusion gene produces chimeric protein and constitutively activates signaling through autophosphorylation. The *KIAA1217-RET51* or *KIAA1217-RET9* fusion construct was cloned and infected with pLenti-*LacZ* (negative control), -*EML4-ALK* (positive control), -*KIAA1217-RET51*, or -*KIAA1217-RET9* virus and stably expressed in BEAS-2B cells. (**E**) Cellular localization of KIAA1217-RET51 or KIAA1217-RET9 fusion proteins. Transformed BEAS-2B cells were loaded onto pre-coated collagen (10 μg/mL) cover slides and incubated for 18 h. After incubation, cells were fixed and stained with anti-RET. Images were taken at × 400 (upper panel) and 800 × (lower panel) magnification. Scale bars represent 20 μm.

To screen for the *RET* fusion partner gene, we prepared a cDNA library from the patient sample and validated the fusion candidate using reverse transcription polymerase chain reaction (RT-PCR) across the fusion breakpoint. The partner gene was confirmed by Sanger sequencing (Figure 1F). According to PCR analysis, genomic recombination occurred between the 553rd nucleotide at *KIAA1217* intron 11 and the 56th nucleotide within *RET* exon 10. After fusion, a portion of *RET* exon 10 was excised through a more complex rearrangement along with *KIAA1217* intron 11, resulting in the novel transcript involving *KIAA1217* exon 11 fused to *RET* exon 11 (Figure 1F). This example illustrates after gene fusion events to produce novel transcripts even if the fusions are happened at the DNA level (Figure 1F). The KIAA1217-RET fusion protein contains both coiled-coil and kinase domains and can be predicted as an oncogenic driver gene such as *KIF5B-RET* (Figure 2A).

### Functional validation of *KIAA1219-RET* fusion gene *in vitro* and *in vivo*

In comprehensive studies of *RET*, the *RET* gene is alternatively spliced in its 3′ region and produces three different isoforms, RET51, RET43, and RET9 that contain 51, 43, and 9 amino acids in their C-terminal region, respectively [26, 27]. RET9 and RET51 have been well studied, as these are the most common isoforms [26, 28], and known to differentially induce biochemical and biological responses [23, 26, 29]. However, the association with RET isoforms and tumorigenic potentials in RET fusion-positive cancer is not clearly known. To investigate RET isoform expression patterns in RET fusion-positive cancer, we screened mRNA expression levels by qRT-PCR using cDNA samples obtained from *RET* fusion-positive lung (Figure 2B and 2C) and thyroid (Supplementary Figure S1) cancer specimens. Gene-expression profiles showed that RET51 was highly expressed in both tissues relative to RET9. In the patient with the *KIAA1217-RET* fusion, RET51 was expressed at much higher levels relative to RET9 (Figure 2C, red circle). Our results indicated that RET51 is primarily expressed in patients having RET fusion-positive tumors.

Comprehensive studies of *RET* describe that *RET* produces at least two isoforms by alternative splicing that have different C-terminal amino acids [30]. RET isoforms displayed distinct gene-expression patterns and promoted different levels of cell differentiation and activation of downstream signals in tumors [23, 26]. To test the tumorigenic potential of *KIAA1217-RET* [both the longer (RET51) and shorter (RET9) RET isoforms], we generated the *KIAA1217-RET51* and *KIAA1217-RET9* fusion constructs and established them in BEAS-2B and NIH3T3 cell lines. Cells harboring *KIAA1217-RET51* or *KIAA1217-RET9* expressed the chimeric protein with a molecular weight of 135 kDa (the longer KIAA1217-RET51) or 130 kDa (the shorter KIAA1217-RET9) and induced ligand-independent phosphorylation (Figure 2D). As described in Figure 1D, KIAA1217-RET51 and KIAA1217-RET9 fusion proteins were mainly localized in cytoplasmic regions but partially expressed in plasma membranes (Figure 2E). This is because the 5′ fusion partner KIAA1217 is mainly localized in cytoplasm, leading to localization of the RET fusion proteins in cytoplasm.

**Figure 3:**
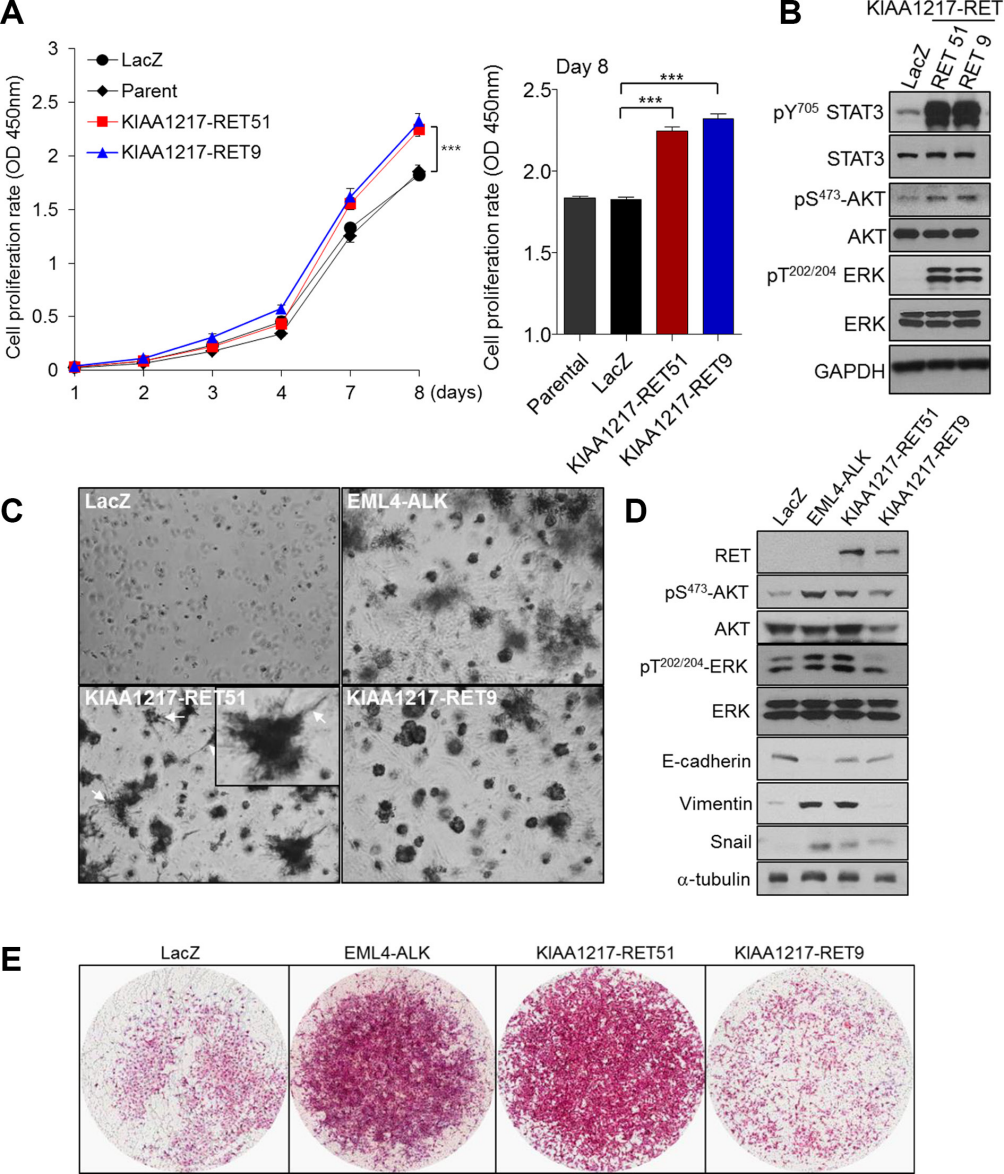
Functional effects of KIAA1217-RET fusion proteins through *in vitro* assays (**A**) Activation of RET-associated downstream molecules following expression of KIAA1217-RET fusion protein. 293FT cells were transfected with the indicated gene, cultured for 48 h, and analyzed by western blots. (**B**) KIAA1217-RET fusion proteins promote cell proliferation. BEAS-2B cells stably expressing indicated protein were seeded into 96-well plates and cultured for 8 days. At the indicated day, absorbance was measured at 450 nm using WST solution. The values shown represent the average of three independent experiments and error bars indicated standard deviations. ***p < 0.0001. (**C**) Images of cells expressing the indicated protein in Matrigel. BEAS-2B cells expressing the indicated fusion protein were cultured for 7 days, with the media refreshed every other day. Images were obtained by phase-contrast microscope at 40 × magnification. (**D**) KIAA1217-RET fusion proteins activate ERK1/2 and AKT, and increase the expression of vimentin and snail in cells. BEAS-2B cells cultured into Matrigel for 7 days were lysed and analyzed for the indicated protein by western blot. (**E**) Invasive abilities of cells expressing KIAA1217-RET fusion proteins. BEAS-2B cells expressing indicated protein were loaded onto transwells pre-coated with Matrigel (1 mg/mL), incubated for 24 h, and invading cells were fixed and stained with hematoxylin and eosin. Images were obtained using a ScanScope XT slide scanner at 10 × magnification.

**Figure 4:**
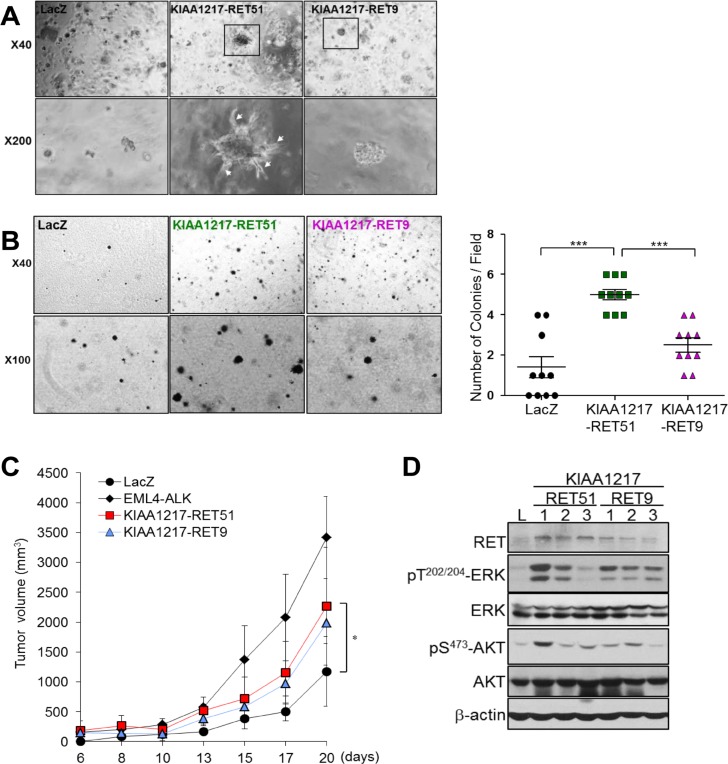
Validation of enhanced tumorigenic activities in cells expressing KIAA1217-RET fusion protein (**A**) *In vitro* transforming assay in Matrigel. NIH3T3 cells stably expressing the indicated protein were cultured in Matrigel for 7 days and the media refreshed every other day. Images were obtained by phase-contrast microscope at the indicated magnification. (**B**) Transformed foci formation in soft agar. NIH3T3 cells expressing the indicated protein in soft agar were seeded in 24-well plates and cultured for 21 days. The number of colonies formed per well was indicated as the mean ± standard deviations from three wells in one representative experiment out of three. ***p < 0.0001 (**C**,**D**) Transforming potential of the KIAA1217-RET fusion protein *in vivo*. NIH3T3 cells expressing the indicated fusion protein were injected into the right dorsal flank of five nude mice. (C) Tumor volume was measured three times/week and calculated using the modified formula. Error bars indicated standard deviations. *p < 0.05. (D)Western blot analysis of xenografted tumors.

RET fusion proteins, such as CCDC6-RET and KIF5B-RET, lead to anchorage-independent cell growth and formation of multiple transformed foci [17]. To validate tumorigenic potential of a KIAA1217-RET fusion, we tested cell growth and tumor-forming ability *in vitro*. Cells expressing the KIAA1217-RET51 or KIAA1217-RET9 fusion protein showed increases in cell proliferation and anchorage-independent growth relative to the LacZ control in BEAS-2B cells (Figure 3A and 3C). Simultaneously, the expression of the KIAA1217-RET fusion protein led to activation of RET downstream signaling molecules, including STAT3, AKT and Erk (Figure 3B). Consequently, KIAA1217-RET fusion led to the increase in tumor cell invasion (Figure 3E). Interestingly, some cells expressing the KIAA1217-RET51 fusion protein showed invasive finger-like protrusions (Figure 3C, white arrows) and expressed mesenchymal markers, such as vimentin and snail in Matrigel-embedded three-dimensional cell cultures (Figure 3D). Similar results were observed in NIH3T3 cells (Figure 4A and 4B). Especially, cells expressing the KIAA1217-RET51 fusion protein formed significantly more colonies than cells expressing the KIAA1217-RET9 fusion protein (Figure 4B). To verify our results, we performed a tumorigenicity assay in a nude mouse (Figure 4C). All five nude mice injected with cells expressing the KIAA1217-RET fusion protein developed a subcutaneous tumor, with the tumor size larger than that observed in the LacZ control (Figure 4C). Additionally, we also observed activation of AKT and ERK in tumors (Figure 4D). Taken together, these data suggest that KIAA1217-RET fusion play a key role for oncogenesis in NSCLC.

### Effect of the RET inhibitor vandetanib on cells expressing KIAA1217-RET

**Figure 5:**
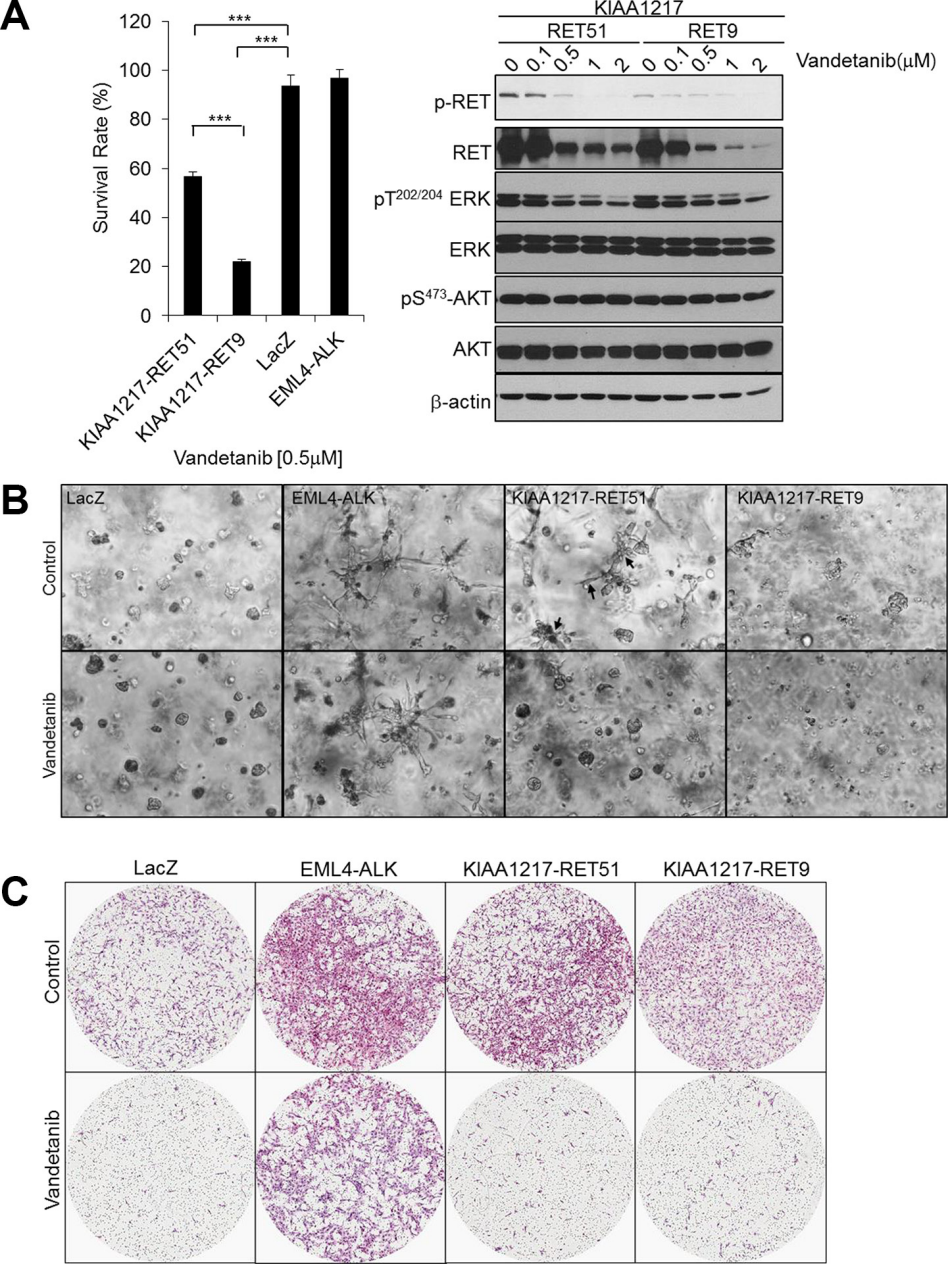
The effects of vandetanib in cells expressing KIAA1217-RET fusion proteins (**A**) Vandetanib reduces proliferation and activation of ERK in cells expressing KIAA1217-RET fusion proteins. (A, left panel) BEAS-2B cells expressing the indicated fusion protein were treated with 0.5 μM vandetanib for 72 h and cell viability was determined. Each bar represents hextuplicate biological replicates ± the standard deviation. ***p < 0.0001. (A, right panel) Cells were treated with the indicated dose of vandetanib for 24 h, followed by cell lysis and detection of the indicated protein by western blot. (**B**) *In vitro* colony-forming ability of KIAA1217-RET-positive cells following vandetanib treatment. BEAS-2B cells were embedded in Matrigel and cultured with or without 0.5 μM vandetanib for 7 days. The images were taken by a phase-contrast microscope at 40 × magnification. (**C**) Invasion abilities of KIAA1217-RET-expressing cells. BEAS-2B cells expressing the indicated protein were loaded onto transwells pre-coated with Matrigel (1 mg/mL), incubated with or without 0.5 μΜ vandetanib for 12 h, and invading cells were fixed and stained with hematoxylin and eosin. The images were obtained using a ScanScope XT slide scanner at 10 × magnification.

RET can be effectively inhibited by several small-molecule tyrosine-kinase inhibitors (TKIs). Currently, two TKIs, the vascular endothelial growth-factor receptor 2 (VEGFR2)- and epidermal growth-factor receptor (EGFR)-inhibitor vandetanib, and the MET- and VEGFR2-inhibitor cabozantinib, are approved by the US Food and Drug Administration for the treatment of locally advanced and metastatic medullary thyroid cancer [9, 13, 21]. Clinical studies in unselected NSCLC-patient populations conducted with RET TKIs did not demonstrate particular survival benefits; however, case reports described promising therapeutic responses to both vandetanib and cabozantinib in patients with RET-fusion-positive lung adenocarcinomas [13, 31, 32]. Therefore, we used a cell-viability assay to investigate the effect of RET-TKIs, such as vandetanib, cabozantinib, and ponatinib, on cells expressing KIAA1217-RET-fusion proteins in order to determine whether the *KIAA1217-RET* fusion could become a novel target of RET inhibitors. Our results showed that cell proliferation rates were reduced by vandetanib treatment of KIAA1217-RET-transformed BEAS-2B cells relative to parental cells (KIAA1217-RET9, IC_50_ = 0.34 μM; parental IC_50_ = 0.67 μM, Supplementary Figure S2A). Additionally, we observed that vandetanib treatment induced dose-dependent inhibition of ERK phosphorylation, but not AKT, in cells expressing the KIAA1217-RET fusion proteins (Figure 5A, right). Ponatinib also inhibited the growth of cells expressing KIAA1217-RET fusion proteins with similar potency, while cabozantinib did not show considerable effective responses (Supplementary Figure S3). The invasive potential following vandetanib treatment of cells expressing KIAA1217-RET fusion proteins was analyzed using three-dimensional Matrigel- and transwell-invasion assays (Figure 5B and 5C). As expected, vandetanib treatment reduced cell growth and invasion and specifically inhibited formation of invasive finger-like protrusions in cells expressing the KIAA1217-RET51 fusion protein (Figure 5B, arrow). Cells transformed with the KIAA1217-RET51 fusion display a more invasive phenotype and exhibit less sensitivity to vandetanib treatment as compared to cells transformed with the KIAA1217-RET9 fusion. To further investigate whether the anti-oncogenic effects of vandetanib were mediated through specific cell cycle arrest in cells expressing KIAA1217-RET fusion protein, we examined cell cycle phase distribution after vandetanib treatment using flow cytometric analysis. Our data showed that 0.5 μM vandetanib treatment resulted in a significant accumulation of G1 phase cells expressing the KIAA1217-RET fusion protein (Supplementary Figure S4). The percentage of cells expressing KIAA1217-RET51 (36.59%) or KIAA1217-RET9 (36.78%) in G1 phase following treatment with 0.5 μM vandetanib markedly increased as compared to cells expressing LacZ (31.62%). These data support that the *KIAA1217-RET* fusion is a novel oncogenic-driver gene and may be a potential target of vandetanib to prevent tumor progression in NSCLC.

## DISCUSSION

**Figure 6:**
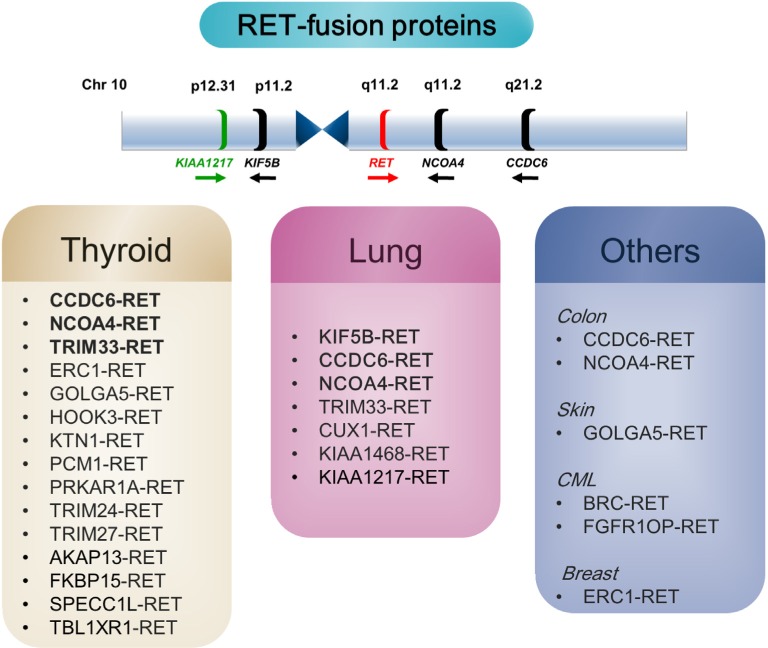
*RET*-fusion genes in cancer (Upper panel) Location of the *RET* oncogene and its fusion partner on chromosome 10. (Lower panel) Schematic summary of the *RET* fusion genes associated with *RET*-positive cancer. The *CCDC6* gene product, also known as *H4 (D10S170), TPC,* or *TST1*, generates the oncogene *RET/PTC1*. *RET/PTC3* is a fusion of *RET* and the activating *NCOA4* gene (*RFG/ELE1/PTC3/ARA70* gene). *GOLGA5* (*PTC5*), generates the oncogene *RET/PTC5*, and *TRIM33* (*ECTO/PTC7/RFG7*) generates the oncogene *RET/PTC7*. TRIM 24/27/33, tripartite motif-containing 24/27/33; ERC1, ELKS/RAB6-interacting/CAST family member 1; GOLGA5, golgin A5; HOOK3, hook microtubule-tethering protein 3; KTN1, kinectin 1; PCM1, pericentriolar material 1; PRKAR1A, protein kinase, cAMP-dependent regulatory type I alpha; AKAP13, A-kinase anchor proteins; FKBP15, FK506-binding protein; SPECC1L, sperm antigen with calponin homology And coiled-coil domains 1-like; TBL1XR1, transducin (beta)-like 1 X-linked receptor 1; BCR, breakpoint cluster region; FGFR1OP, FGFR1 oncogene partner.

*RET* fusion genes in lung adenocarcinomas appear predominantly following intrachromosomal rearrangement. In a previous report, we detected 15 *RET* fusion transcripts in cells taken from a lung cancer patient and confirmed five *RET* fusion partners (*KIF5B, CCDC6, TRIM33, NCOA4, and CUX1*) [7]. All of these genes are located on chromosome 10, except for *TRIM33* and *CUX1* (Figure 6). This study is the first report of a novel *KIAA1217-RET* fusion gene in NSCLC. The *KIAA1217* gene is also located on the short arm of chromosome 10 (10p12.2 locus), similar to *KIF5B* (10p11.22), which is the most common *RET* partner gene in NSCLC [33], and fused with *RET* by chromosomal rearrangements.

*KIAA1217* is a novel *RET* partner gene in lung cancer and contains two CCDs that could promote ligand-independent dimerization in the RET fusion protein [34]. Previous researches reported that CCDs in fusion partners associated with tyrosine-kinase fusion proteins can induce an aberrant activation of the kinase domain through ligand-independent dimerization, as observed in ALK-fusion proteins [9, 11, 19, 35–38]. Additionally, the BioGPS-based mRNA-expression profile showed that KIAA1217 is ubiquitously expressed across a variety of human tissues, and that its promoter can lead to high transcriptional activity. Given these findings, we suggest that the novel KIAA1217-RET fusion generates an aberrant chimeric protein and consequently gives rise to oncogenic potentials for tumorigenicity. Here, we demonstrated these activities through *in vivo* and *in vitro* assays using transformed BEAS-2B and NIH3T3 cells (Figures 3 and 4).

To date, RET specific inhibitors have not been fully validated in clinical trials and there is no established therapy for RET fusion-positive patients [39, 40]. In this study, our data indicated that cells expressing the KIAA1217-RET fusion protein were susceptible to vandetanib treatment, which decreased cell proliferation via reduction of ERK phosphorylation (Figure 5A). Although previous studies suggested that cabozantinib treatment exhibited greater inhibitory activity relative to vandetanib treatment in cells harboring the *RET/papillary thyroid carcinoma (PTC)* fusion gene, which is also known as *CCDC6-RET*, [41, 42] and preliminary data from Drilon et al. [13] revealed partial responses to cabozantinib treatment in a prospective phase II trial involving patients with *RET*-fusion-positive NSCLC, cabozantinib appears to be an ineffective agent for cells expressing KIAA1217-RET fusion proteins (Supplementary Figure S4). This is likely due to TKI treatment of RET positive cancer exhibiting partial and different responses against aberrant forms of RET according to its fusion partners [13, 41].

Alternative splicing of RET results in two main isoforms, RET9 and RET51, which differ in their C-terminal regions. Previous studies reported that RET51 and RET9 are both expressed in most tissues [43] and that RET9 is frequently expressed at higher levels relative to RET51 in human pheochromocytomas [26]. However, here, we observed that RET51 is the more frequently expressed form relative to RET9 in most RET fusion-positive samples. This phenomenon is thought to occur since this study is performed in RET fusion-positive patient group in NSCLC and it might mean that RET fusion partners in NSCLC preferably contains the longer transcript since it is more aggressive and evolutionarily selected during cancer progression. We also showed that the RET51 isoform of KIAA1217 fusion exhibited a more aggressive phenotype and invasive potential along with less sensitivity to vandetanib [9]. This is likely due to RET51 having an additional phosphorylation site (Y1096), which is a key docking site that binds to SH2-domain-containing proteins, including growth factor receptor-bound protein 2 (GRB2), which leads to the recruitment of additional adaptors (GRB2-associated binding protein 1 or 2). This stimulation of multiple signaling pathways promotes downregulation of RET51 [6, 23].

Here, we identified a novel *KIAA1217-RET*-fusion gene, described its oncogenic function and pro-invasive abilities, and demonstrated differences in vandetanib sensitivity between the two resulting RET isoforms. The results of this report are summarized in Figure 6, illustrating the differences in the *RET*-fusion partner genes involving *KIAA1217*. This study provided experimental evidence supporting the use of vandetanib and promoting *KIAA1217-RET* as a novel target for personalized cancer therapy. The results presented here allow for the expansion of treatment options through detection of novel fusion genes in unselected NSCLC patients.

## MATERIALS AND METHODS

### Patient, cell lines, and reagents

The patient was a 51-year-old Korean woman with a non-smoking history who underwent a lobectomy due to an occurrence of an irregular mass on the right-lower lobe. The mass was confined to the lung parenchyma, did not involve the visceral pleura, and no metastasis was found in 27 lymph nodes. After 4 years of follow-up, computed tomography scans did not reveal any evidence of recurrence or metastasis in the patient without adjuvant treatment. This study was approved by the Institutional Review Board of the Samsung Medical Center. The bronchoepithelial cell line BEAS-2B (human bronchial epithelium) was obtained from the American Type Culture Collection (ATCC CRL-9609; Manassas, VA, USA), and NIH3T3 cells and 293FT cells were obtained from the Korean Cell Line Bank (Seoul, Korea). The cells were cultured in Dulbecco's Modified Eagle Medium (DMEM; JBI, Daegu, Korea) supplemented with 10% fetal bovine serum (FBS; Life Technologies, Carlsbad, CA, USA), 100 U/mL penicillin, and 100 mg/mL streptomycin (Life Technologies). Vandetanib, cabozantinib, and ponatinib were obtained from Selleck Pharmaceutical (Houston, TX, USA). Ten *RET*-fusion-confirmed lung adenocarcinomas [7] and 12 thyroid-cancer tissues were collected from the Department of Pathology, Samsung Medical Center (Seoul, Korea) for investigation of RET-isoform expression patterns. All of the cell lines used were authenticated via short tandem-repeat profiling before beginning a new series of experiments and were kept in culture for < 3 months.

### RET immunohistochemistry and FISH

Formalin-fixed paraffin-embedded (FFPE) sections were incubated in a solution of 0.3% H_2_O_2_ for 15 min to inhibit endogenous peroxidase activity. The sections were then incubated for 1 h at room temperature with primary antibody solutions: RET antibody (ab134100; Abcam, Cambridge, UK; 1:200 dilution). The EnVision Systems for rabbit antibodies (K4003; DAKO, Glostrup, Denmark) were applied according to manufacturer instructions. Slides were stained with liquid diaminobenzidine tetrahydrochloride, a high-sensitivity substrate-chromogen system (K3468; DAKO). *RET* FISH tests were performed on FFPE tumor tissues using ZytoLight SPEC RET Dual Color Break Apart Probes according to manufacturer instructions (ZytoVision, Bremerhaven, Germany). The SPEC RET Dual Color Break Apart Probe is a mixture of two direct-labeled probes hybridizing to the 10q11.21 band. The orange fluorochrome direct-labeled probe hybridizes proximal to the *RET* gene and the green fluorochrome direct-labeled probe hybridizes distal to the gene. Signals for each locus-specific FISH probe were assessed under an Olympus BX51TRF microscope (Olympus, Tokyo, Japan) equipped with a triple-pass filter (4′,6-diamidino-2-phenylindole/Green/Orange; Vysis, Downers Grove, IL, USA).

### cDNA library for the *KIAA1217-RET* fusion

Total mRNA was extracted from the tumor tissue using an RNeasy FFPE Kit (Qiagen, Hilden, Germany) according to manufacturer instructions. To obtain a cDNA library to screen fusion transcripts involving *RET* as a fusion partner, a double-stranded cDNA fragment corresponding to the conjoined region of the *KIAA1217–RET*-fusion transcript was amplified by SMARTer 5′ rapid amplification of cDNA ends cDNA Amplification Kit (Clontech Laboratories, Inc., Mountain View, CA, USA). The PCR products were then cloned into a pCR4-TOPO vector using the TOPO TA Cloning Kit (Invitrogen, Carlsbad, CA, USA) for DNA sequencing. The cDNA sequence of the novel *KIAA1217-RET*-fusion gene has been deposited in the National Center for Biotechnology Information database under accession numbers NM_001282767.1, NM_020975.4, and NM_020630.4.

### Cloning full-length *KIAA1217-RET51 and KIAA1217-RET9* cDNA

For the *KIAA1217-RET9* construct, cDNA was generated by amplifying two separate overlapping *KIAA1217* and *RET* fragments that were generated by overlap-extension PCR. For the first PCR, the *KIAA1217* fragment was amplified with the following primer sets from MCF10A: Forward, KIAA1217 F, 5′-CCAGAGAGCGAGGAGCTTT-3′; Reverse, KIAA1217 R (including breakpoints), 5′-GTCGCACAGT GGATCTTTCAGGGTAG CTACAGCCTC-3′. The *RET9* fragment was generated from pDORN-RET (addgene #23906) with the following primer sets: Forward, RET51 F(including breakpoints), 5′-GTAGCTACCCTGAA AGATCCACTGTGCGACGAGCT-3′; Reverse, RET9 endR, 5′-GAATCTAGTAAATGCATGG GAAATT-3′. The second PCR was performed using a mixture of the first PCR products with the KAA1217 F and RET9 endR primers. The PCR for the RET51 fragment was performed using the cDNA from the tumor specimen harboring the *KIAA1217-RET* fusion with the following primer sets: Forward, RET 3063F, 5′-CACTCCATCTGACTCCCTGATT-3′; RET51 endR, 5′-ACTATCAAACGTGTCCATTAA TTTTG-3′. For the *KIAA1217-RET51* fusion construct, the *KIAA1217* fragment was generated from the *KIAA1217-RET9* construct and used as the template with the following primer sets: Forward, KIAA1217 F; Reverse, RET 3092R, 5′-TCGTCATAAATCA GGGAGTCAGAT-3′. The second overlapping PCR was performed with the KIAA1217 F and RET51 endR primers. The full-length *KIAA1217-RET* PCR products were ligated into the gateway entry vector pCR8/GW/TOPO (Invitrogen, Life Technologies) and then transferred into the pLenti6.3/V5-DEST gateway vector (Invitrogen, Life Technologies) according to manufacturer instructions.

### Western blots

The cell lysates from cells or tissues expressing LacZ, EML4-ALK (GenBank: AB663645.1), KIAA1217-RET51, or KIAA1217-RET9 were prepared with a modified radioimmunoprecipitation assay buffer (50 mM Tris-HCl, 150 mM NaCl, 1% NP-40, and 0.25% sodium deoxycholate) plus a protease-inhibitor cocktail (GenDepot, Katy, TX, USA). The lysates were centrifuged at 15,000 g for 30 min at 4°C, diluted with 4X sample buffer, and boiled for 10 min. The samples were separated on 8–10% sodium dodecyl sulfate-polyacrylamide gel electrophoresis gels and transferred to a polyvinylidene difluoride membrane (Immobilon P; EMD Millipore, Darmstadt, Germany). Blots were blocked using 5% skim milk (BD Difco; BD Biosciences, East Rutherford, NJ, USA) and incubated the following antibodies at 4°C overnight; Phosphor-RET(Tyr905, #3221), STAT3(Tyr706, #9145), AKT(Ser473, #4060), ERK(Thr202/Tyr204, #4370) antibodies and total-RET(#3223), STAT3(#4904), AKT(#2920), ERK(#9102), vimentin, E-cadherin, and snail (Epithelial-Mesenchymal Transition (EMT) Antibody Sampler Kit, #9782) antibodies (Cell Signaling Technologies, Danvers, MA, USA), or α-tubulin(#8035) or GAPDH(#25778) antibodies (Santa Cruz Biotechnology, Inc., Santa Cruz, CA, USA). Blots were washed and incubated with the secondary antibodies and detected using a chemiluminescent horseradish peroxidase substrate (EMD Millipore). All western-blot images are representative of at least three independent experiments.

### Immunofluorescence

Immunofluorescence was performed using the following process. Glass coverslips were coated with 10 μg/mL collagen (#354236; BD Biosciences), and cells expressing the indicated gene or an empty vector were seeded on pre-coated coverslips. After 24 h, cells were fixed in 4% paraformaldehyde and permeabilized with 0.5% Triton X-100 in phosphate-buffered saline (PBS). Cells were incubated with 5% FBS in PBS for 1 h and then incubated at 4°C with anti-RET antibody (#3223, Cell Signaling Technology). After overnight incubation, cells were incubated with Alexa-fluor 488 rabbit anti-goat secondary antibody (Molecular Probes; Life Technologies), washed three times with PBS, and mounted in Vectashield mounting medium (Vector Laboratories, Peterborough, UK). The images were taken with a Zeiss LSM 750 confocal microscope (Carl Zeiss, Oberkochen, Germany).

### Cell proliferation (viability) assay

Cell proliferation rates were measured with an EZ-Cytox cell viability assay kit (Daeil Lab Service, Seoul, Korea) according to manufacturer instructions. BEAS-2B (2 × 10^3^) or NIH3T3 cells (1 × 10^3^) expressing the indicated gene or an empty vector were seeded into 96-well plates. On the indicated day, 10 μL of EZ-Cytox reagent was added to each well and incubated for 2 h at 37°C. The absorbance was measured at 450 nm (foreground) and 650 nm (background) using a spectrophotometer. IC_50_ values were calculated using GraphPad Prism 5 (v5.01; GraphPad Software, Inc., La Jolla, CA, USA).

### Relative quantitation of RET51 or RET9 using real-time PCR

For all tissue samples, total cellular RNA was extracted from FFPE tissue with RNeasy FFPE Kit (Qiagen) according to manufacturer instructions. The RET transcripts were amplified by real-time PCR using RET specific variant-51 or variant-9 primers; RET 51 forward, 5′-ACGAGAGCTGATGGCACTAA-3′; RET 51 reverse, 5′-TGAGGGTGAAAGCATCCAGT-3′; RET 9 forward, 5′-CCGCTGGTGGACTGTAATAATG-3′; RET9 reverse, 5′-CTAGTAAATGCATGGGAAATTCTAC-3′. Standard curves were obtained by plotting crossing threshold (Ct) values using absolute quantitative real-time PCR (qRT-PCR) for serially diluted plasmid DNA containing RET 51 or RET 9 over a 10^5^-fold range. To correct the differences in RET transcript amplification efficiency using the different primers, constant-amplification efficiency was calculated from the slope on the basis of ΔΔCt values using assumptions of log-linear analysis. Relative expression levels of RET 51 were normalized using the constant-amplification efficiency of RET 9. Then, the relative RET9 and RET51 levels were calculated using the following equation: RET51 = RET × {RET51 × (RET9+RET51)}, RET9 = RET × {RET9 × (RET9+RET51)}.

### *In vitro* transforming assay

Analysis of the transforming activity of kinase fusions was performed with Matrigel (BD Biosciences) and soft agar. NIH3T3 or BEAS-2B stable cells expressing LacZ, EML4-ALK, KIAA1217-RET51, or KIAA1217-RET9 were cultured in Matrigel. The bottom layer of each well was coated with 30 μL of Matrigel and allowed to gel by incubating for 30 min at 37°C. Then, 10,000 cells resuspended in 150 μL of Matrigel were loaded onto the bottom layer of each well. Medium with 10% FBS was then overlaid onto the gel and replaced every other day. The images of transformed foci were obtained after culturing for 7 days. For the soft agar assay, the base layer of each well consisted of 1.5 mL of medium with a final concentration of 0.5% Noble agar (BD Sciences). After bottom agar solidification, 1.5 mL of 0.35% agar containing NIH3T3 cells (20,000) was seeded on the bottom agar layer and incubated for 21 days. Medium was changed every 4 days for 3 weeks. Colonies were fixed with 4% paraformaldehyde and then stained with 0.05% crystal violet (Sigma-Aldrich, St. Louis, MO, USA), and representative images were taken by a phase-contrast microscope (Olympus CKX41) using i-Solution Lite image analysis software (Image & Microscope Tech.). Statistical significance was analyzed by the GraphPad Prism software (v5.01; GraphPad Software, Inc.).

### Xenograft tumor-formation assay

All animal experiments were approved by the Institutional Review Board of Samsung Medical Center. Cells (5 × 10^6^) were resuspended in PBS (pH 7.4) and mixed with an equal volume of high-concentration Matrigel. The mixture was subcutaneously injected into the right dorsal flank of 6-week-old male nude mice (Orient Bio, Gyeonggi-Do, Korea). Mice were monitored three times weekly until tumor size reached approximately 2 × 2 cm. Using electronic calipers, the greatest longitudinal diameter (length) and the greatest transverse diameter (width) were measured, and tumor volume was calculated using the modified ellipsoidal formula [tumor volume = 1/2 (length × width^2^)]. Mice were then sacrificed and photographed on day 23. Statistical significance was analyzed using GraphPad Prism software (v5.01; GraphPad Software, Inc.).

### Invasion assay

Cellular invasion ability was evaluated by using 24-well transwell chambers (Corning Costar #3422; Corning Life Sciences, Durham, NC, USA) with 8 μm polycarbonate membrane filters. Upper chambers contained 20 μL of 1 mg/mL Matrigel in the center of each well and were allowed to spread over the entire surface of the membrane, which was incubated for 2–3 h to allow Matrigel solidification. Cells were incubated in serum-free media for 4 h, harvested, and loaded at 1 × 10^5^ cells/well into upper chambers that had been coated with Matrigel and incubated with or without 2% FBS/DMEM plus 0.5 μM vandetanib. The lower chambers were filled with 10% FBS/DMEM. After an 18-h incubation, the non-migrating cells in the upper chamber were removed with cotton swabs. The migrating cells remaining on the bottom portion of the membranes were fixed with 4% paraformaldehyde and stained with hematoxylin and eosin. The membrane was mounted and images were taken by the Aperio ScanScope XT slide scanner (Aperio Technologies, Inc., Vista, CA, USA) at 10 × magnification.

## SUPPLEMENTARY MATERIALS FIGURES


