# Supplementary material for: Identification of a novel partner gene, KIAA1217, fused to RET: Functional characterization and inhibitor sensitivity of two isoforms in lung adenocarcinoma
Source: Oncotarget. 2016 May 2;7(24):36101–14. doi: 10.18632/oncotarget.9137 (PMC5094986; doi:10.18632/oncotarget.9137)
Supplement: Supplementary file 1 [file oncotarget-07-36101-s001.pdf]

Identification of a novel partner gene, *KIAA1217*, fused to *RET*: Functional characterization and inhibitor sensitivity of two isoforms in lung adenocarcinoma

Supplementary Materials

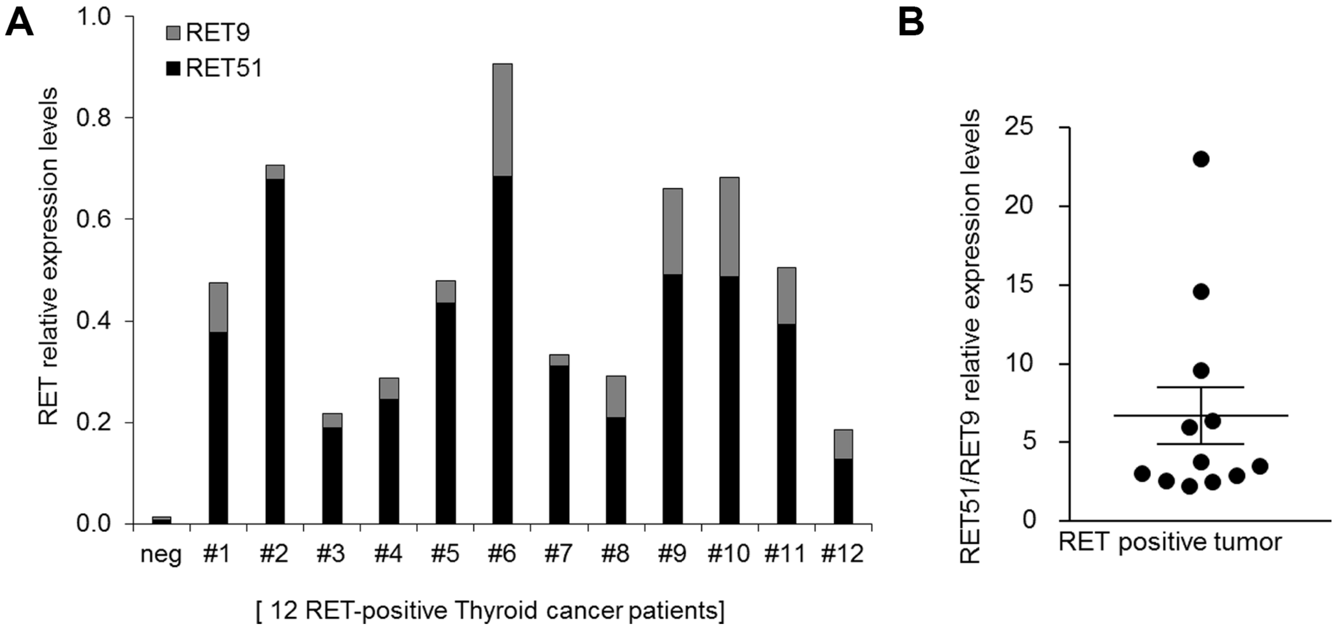

Supplementary Figure S1: Identification of RET isoforms in RET-fusion-positive thyroid tumors. (**A**) Expression levels of total RET, RET9, and RET51 and (**B**) the relative RET51/RET9 ratios from RET fusion-positive thyroid patients.

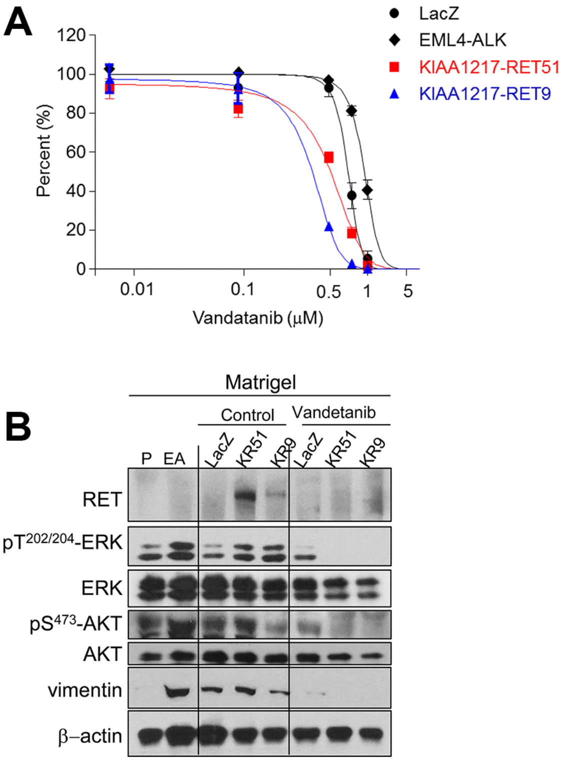

Supplementary Figure S2: The effects of vandetanib in KIAA1217-RET positive cells. (**A**) Growth inhibition by vandetanib treatment. BEAS-2B cells expressing the indicated protein were seeded into 96-well plates, treated with the indicated dosages for 72 h, and analyzed for cell proliferation using WST solution. (**B**) Decrease in ERK1/2 activation and vimentin expression by vandetanib treatment in KIAA1217-RET positive cells. BEAS-2B cells expressing the indicated protein embedded in the Matrigel were cultured for 7 days with or without 0.5 μM vandetanib, lysed, and analyzed by western blot.

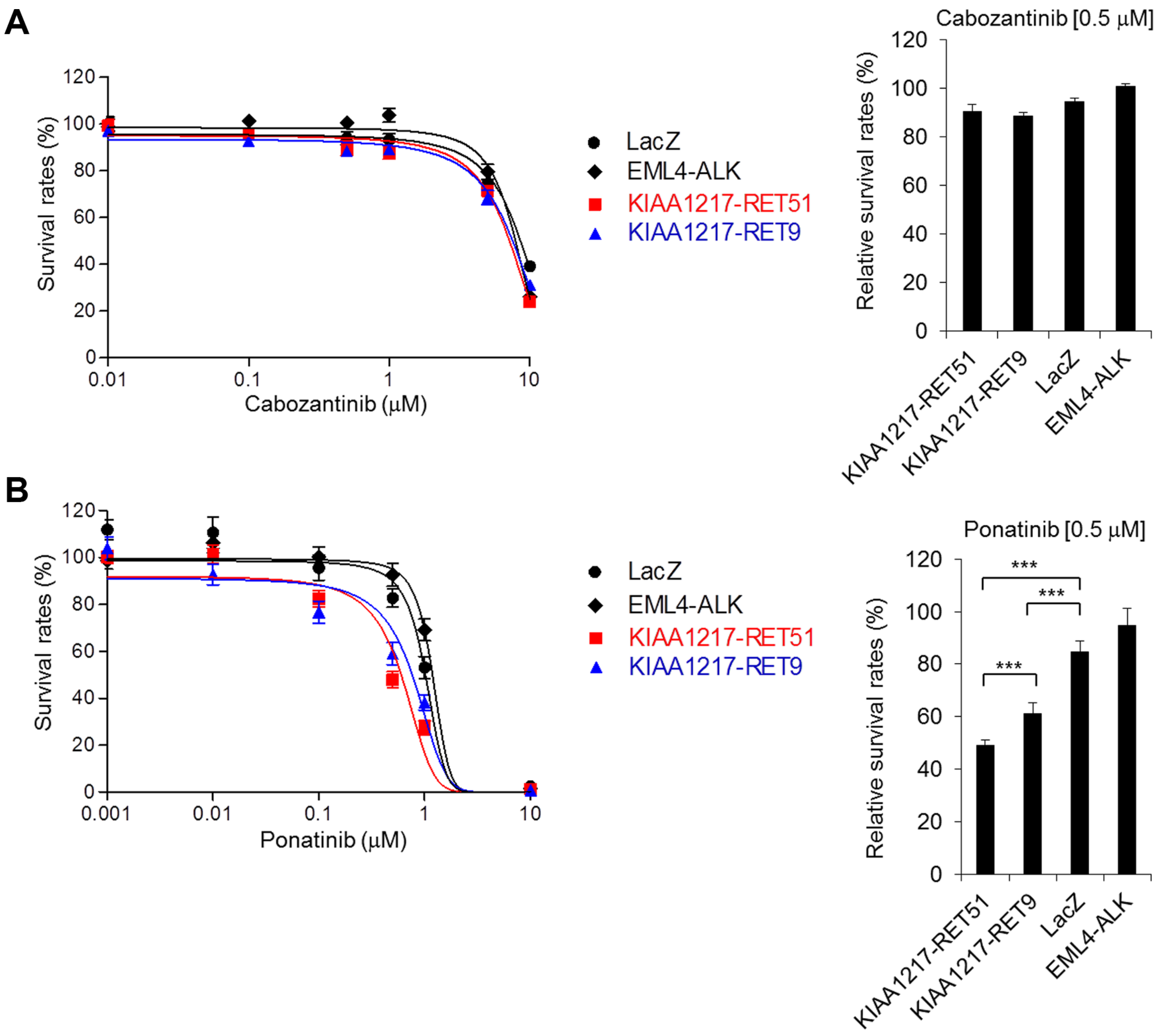

Supplementary Figure S3: The effects of RET-TKIs on KIAA1217-RET-expressing BEAS-2B cells. Growth inhibition following (**A**) carbozantinib or (**B**) ponatinib treatment. BEAS-2B cells expressing the indicated protein were seeded into 96-well plates, treated with the indicated dosages for 72 h, and analyzed for cell proliferation using WST solution. The *p*-values were calculated using an unpaired *t* test. ***$p < 0.0001$.

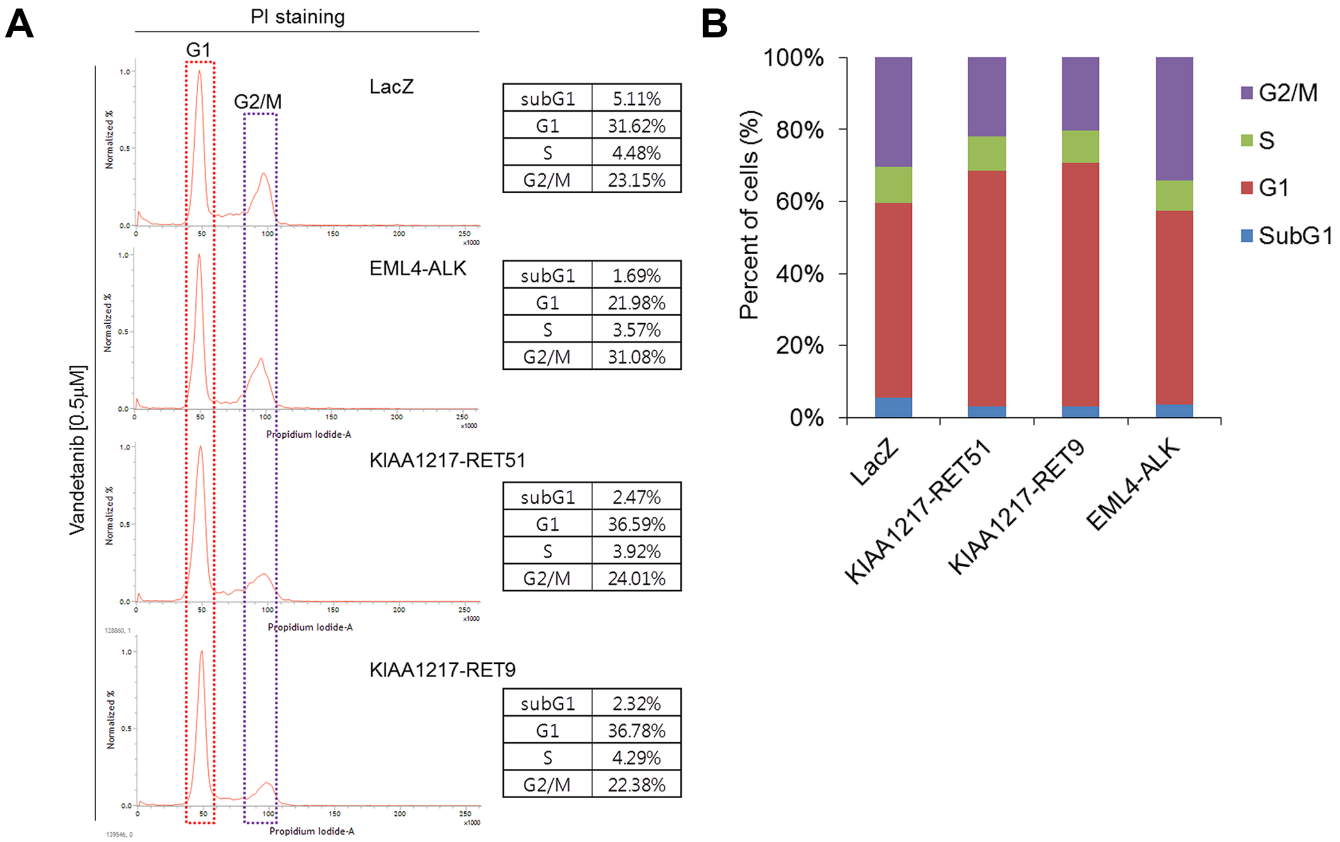

Supplementary Figure S4: Vandetanib treatment causes significant accumulation of G1-phase BEAS-2B cells expressing KIAA1217-RET-fusion protein. (**A**) Representative flow cytometry analysis of cell cycle distribution in the indicated cell lines, which were released to induce G1-phase cell cycle arrest by vandetanib. (**B**) Cell cycle distribution in the cell lines was determined by flow cytometric analysis. The total population of cells in each phase of the cell cycle is shown as the mean ± the standard deviation of three independent experiments.

Cell cycle analysis

For cell cycle analysis following vandetanib treatment, cells were harvested after incubating in the presence of vehicle (dimethyl sulfoxide) or vandetanib (0.5 μM) for 24 h. Cells were centrifuged to obtain a pellet and resuspended in 500 μL PBS with 1% bovine serum albumin. Cell pellets were washed with 4°C phosphate-buffered saline (PBS) and fixed with ice-cold 95% ethanol containing 0.05% Triton X-100 at 4°C. Then, 1 mL PBS containing 50 μg/mL propidium iodide (P4170; Sigma-Aldrich, St. Louis, MO, USA) and 0.5 g/mL RNaseA was added to the cell pellets and incubated for ~30 min at 37°C in the dark. Fluorescence was determined immediately and analyzed with a flow cytometry analyzer (BD Biosciences, East Rutherford, NJ, USA). This assay was performed in triplicate.
